# Transcriptome and Metabolome Analyses Reveal the Mechanism of Corpus Luteum Cyst Formation in Pigs

**DOI:** 10.3390/genes14101848

**Published:** 2023-09-23

**Authors:** Jiage Dai, Jiabao Cai, Taipeng Zhang, Mingyue Pang, Xiaoling Xu, Jiahua Bai, Yan Liu, Yusheng Qin

**Affiliations:** 1 Institute of Animal Husbandry and Veterinary Medicine, Beijing Academy of Agriculture and Forestry Sciences, Beijing 100097, China; djg730@126.com (J.D.); c18833075975@163.com (J.C.); jasmine769@163.com (M.P.); xu_xiaoling1980@163.com (X.X.); bai_jiahua@126.com (J.B.); liuyanxms@163.com (Y.L.); 2 College of Animal Sciences and Technology, China Agricultural University, Beijing 100193, China; 3 College of Life Sciences and Food Engineering, Hebei University of Engineering, Handan 056038, China; ztp5331365@163.com; 4 Animal Science and Technology College, Beijing University of Agriculture, Beijing 102206, China

**Keywords:** luteal cyst, choline, Pi3k-Akt, MAPK, ALDH

## Abstract

Corpus luteum cysts are a serious reproductive disorder that affects the reproductive performance of sows. In this study, transcriptome and metabolome datasets of porcine normal and cyst luteal granulosa cells were generated to explore the molecular mechanism of luteal cyst formation. We obtained 28.9 Gb of high−quality transcriptome data from luteum tissue samples and identified 1048 significantly differentially expressed genes between the cyst and normal corpus luteum samples. Most of the differentially expressed genes were involved in cancer and immune signaling pathways. Furthermore, 22,622 information-containing positive and negative ions were obtained through gas chromatography−mass spectrometry, and 1106 metabolites were successfully annotated. Important differentially abundant metabolites and pathways were identified, among which abnormal lipid and choline metabolism were involved in the formation of luteal cysts. The relationships between granulosa cells of luteal cysts and cancer, immune-related signaling pathways, and abnormalities of lipid and choline metabolism were elaborated, providing new entry points for studying the pathogenesis of porcine luteal cysts.

## 1. Introduction

The normal structure and function of ovaries are very important for the estrus cycle and ovulation in sows. Ovarian cysts are a serious reproductive disorder in sows, and because the clinical symptoms are mild, affected sows are easily overlooked during production, resulting in decreased productivity [1].

The factors involved and the mechanism of luteal cyst formation are complicated [2,3,4]. In the normal estrous cycle of sows, the luteinizing hormone peak causes ovulation, and the ovulating follicles form the luteum and secrete progesterone to maintain the pregnancy. If there is no pregnancy, the corpus luteum dissolves around the sixteenth day of the estrus cycle and the next estrus cycle begins. Disruption of the hypothalamus–pituitary–gonadal axis and disturbance of steroid and gonadotropin receptor expression in the endocrine signaling pathway can lead to cyst formation [5,6,7,8]. Stress, metabolic disorders, and proliferation/apoptosis can also lead to cyst formation [9].

The pathogenesis of luteal cysts is a complicated process that has not been thoroughly investigated, and the specific mechanism is still unclear. The protein and gene expression patterns of follicular granulosa cells are different at different stages [10]. We speculated that the transcriptome and metabolome levels may be different between granulosa cells in cystic luteum and normal luteum. This study may provide a new entry point for studying the pathogenesis of porcine luteal cysts through transcriptome and metabolome analyses, as well as theoretical guidance for the prevention of luteal cysts.

## 2. Materials and Methods

### 2.1. Sample Collection

**Figure 1:**
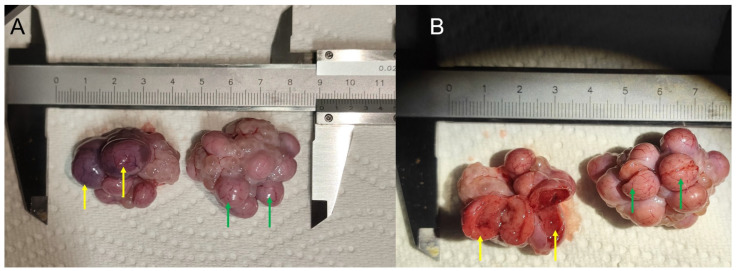
Corpus luteum. Cystic corpus luteum (**A**) and normal corpus luteum (**B**). Corpus luteum cysts ((**A**) yellow arrows) and normal corpus luteum ((**A**) green arrows); Vertical section through corpus luteum cysts ((**B**) yellow arrows) and through normal corpus luteum ((**B**) green arrows).

Cystic corpus luteum and normal corpus luteum were obtained from large-scale commercial pig farms in Beijing (Figure 1). Long−term non−estrus sows were tested through B−ultrasound, identified by professionals as having corpus luteum cysts, and slaughtered. The collected corpus luteum was placed in a 37 °C saline solution containing penicillin−streptomycin solution and transported to the laboratory. The outer membrane of the luteum was peeled off with sterile tooth tweezers. An appropriate amount of luteum tissue was biopsied using eye tweezers, placed in a sterile centrifuge tube, quickly frozen with liquid nitrogen, and transferred to a −80 °C refrigerator. Normal corpus luteum was collected, processed, and stored in the same way.

### 2.2. RNA Extraction

RNA was extracted from the tissue samples using TRIzol reagent according to the manufacturer’s instructions (Invitrogen, Carlsbad, CA, USA). The RNA integrity was assessed using 1% agarose gel assays. The RNA concentration was measured using a Nanodrop microspectrophotometer, and the sample quality was determined using an Agilent 2100 electrophoretogram (Agilent Technologies, Palo Alto, CA, USA).

### 2.3. Library Preparation and Sequencing

We used 3 µL mRNA from each sample and enriched it for poly(A) mRNAs using oligo-dT magnetic beads (Epicentre, Madison, WI, USA). The mRNAs were disrupted through ultrasound, and first-strand cDNA was synthesized using an M-MuLV reverse transcriptase system (NEB# M0253L; New England Biolabs, Ipswich, MA, USA) with six-base random primers. Second-strand cDNA was synthesized using endonuclease (RNaseH) and a DNA polymerase I system. After purification, end repair, and ligation of the sequencing adapters, cDNA that was approximately 200-bp long was screened for using AMPure XP beads (Agencourt, Beckman Coulter, Brea, CA, USA). After amplification, the cDNA was purified with AMPure XP beads, and PCR products were obtained to complete the library. The library concentration and size of the inserted fragment were tested using Qubit 2.0 (Invitrogen, USA) and an Agilent 2100 Bioanalyzer (Agilent Technologies, CA, USA). Agarose gel electrophoresis and a nanophotometer spectrophotometer were used to measure the RNA integrity and purity, after passing the library inspection. To obtain high-quality data, we removed reads that contained adapters, reads with >10% N bases (all of them were A bases), and/or reads with Q ≤ 20 bases, which made up >50% of the read sequence. RNA sequencing of the resultant clean reads was performed on an Illumina-HiSeq X sequencing platform with a read length of 150 bp.

### 2.4. Identification of Single Nucleotide Polymorphisms (SNPs)

After quality control, the sequencing data were compared with the pig reference genome (*Sus scrofa* isolate TJ Tabasco breed Duroc, GCF_000003025.6_Sscrofa11.1). The reads were mapped to chromosome coordinates, duplicate reads were removed, and reads with mass values < 30 were filtered out. The Genome Analysis Toolkit (GATK) was used to detect SNP loci [11]. SNPs related to differentially expressed genes (DEGs) were analyzed to identify genes that were potentially related to the occurrence of luteal cysts.

### 2.5. Analysis of Differentially Expressed Genes

The DESeq2 software [12] was used to analyze the DEGs, and |log2 (fold change)| > l and FDR < 0.05 were used as the screening criteria. The selected DEGs were annotated using the Gene Ontology (GO; http://geneontology.org/, accessed on 5 April 2022) and Kyoto Encyclopedia of Genes and Genomes (KEGG) databases (http://www.genome.jp/kegg/, accessed on 5 April 2022).

### 2.6. Screening and Quantitative Fluorescence Analysis of Luteal Cyst Genes

The KEGG pathways were ranked based on q−values; low q−values indicated significant differences between the corresponding pathways. The selected DEGs were verified through quantitative PCR (qPCR), and the expression patterns of genes that were differentially expressed between normal and cyst luteal granulosa cells were analyzed. The primer sequences were synthesized by Shanghai Bioengineering Co., Ltd. (Shanghai, China) and are presented in Supplementary Table S1. Quantitative amplification of the cDNA was performed using an iScript kit (Bio−Rad Laboratories, Hercules, CA, USA). The Bio−Rad Chrome real-time qPCR system was used to perform qPCR, and the 2^−ΔΔCT^ method was used to calculate the relative expression level of each gene. Each group had three technical replicates and three biological replicates.

### 2.7. Metabolite Identification and Differential Metabolite Analysis

Vanquish ultra-high performance liquid chromatography (UHPLC) (Thermo Fisher Scientific, Waltham, MA, USA) and an Orbitrap Q Exactive TM HF−X mass spectrometer (Thermo Fisher Scientific, Sunnyvale, CA, USA) were used for liquid chromatography tandem mass spectrometry (LC−MS/MS) analysis [13]. The acquired LC-MS raw data were processed using Compound Discover 3.1 (CD3.1, Thermo Fisher Scientific), and retention time, mass-charge ratio, and other parameters were screened. Peak alignment of the different samples was performed using a retention time deviation of 0.2 min and mass deviation of 5 ppm, to increase the accuracy of the identification. Peaks were extracted according to the set mass deviation of 5 ppm, signal strength deviation of 30%, signal-to-noise ratio of 3, minimum signal strength of 100,000, as well as ion and other information. The peak area was quantified, and the target ion was integrated. The molecular formula was predicted using molecular ion peak and fragment ion, and compared with the mzCloud (https://www.mzcloud.org/, accessed on 5 April 2022), mzVault, and Masslist databases [14]. Background ions were removed using blank samples, and the quantitative results were normalized to obtain the identification and quantitative results. The hypothesis test value (*p*-value) of potential differentially abundant metabolites was obtained using Student’s *t*-test, and the fold change values of metabolites were calculated by comparing the average value of each peak area.

### 2.8. Analysis of Differentially Abundant Metabolite Pathways

Differentially abundant metabolite pathways were annotated through enrichment and topological analyses. The Chemical Entities of Biological Interest (http://www.ebi.ac.uk/chebi/init.do/, accessed on 5 April 2022), National Institute of Standards and Technology (http://www.nist.gov/index.html/, accessed on 3 April 2022), KEGG (http://www.genome.jp/kegg/, accessed on 6 April 2022), and other databases were used to identify metabolites, explain the biological functions, and build pathways. MetaboAnalyst 5.0 (http://www.metaboanalyst.ca/MetaboAnalyst/, accessed on 3 April 2022) was used to analyze the different metabolites in the KEGG pathways. The KEGG and PubChem databases were used to determine whether there were significant changes in the pathways that involved cyst and normal corpus luteum genes, and to evaluate the potential effects of metabolite concentrations on specific pathways based on the location of the metabolites in the pathways. The aim was to identify key enzymes and rate-limiting enzymes that play important roles in the significant differentially abundant metabolic pathways.

### 2.9. Combined Metabolomic and Transcriptomic Analyses

Based on the gene expression and metabolite content data, KEGG pathway maps were annotated for differentially abundant metabolites and differentially expressed genes to obtain common synthesis pathway information.

## 3. Results

### 3.1. Transcriptome Sequencing, Statistical Assessment of Sequence Quality, and Expression Analysis

**Figure 2:**
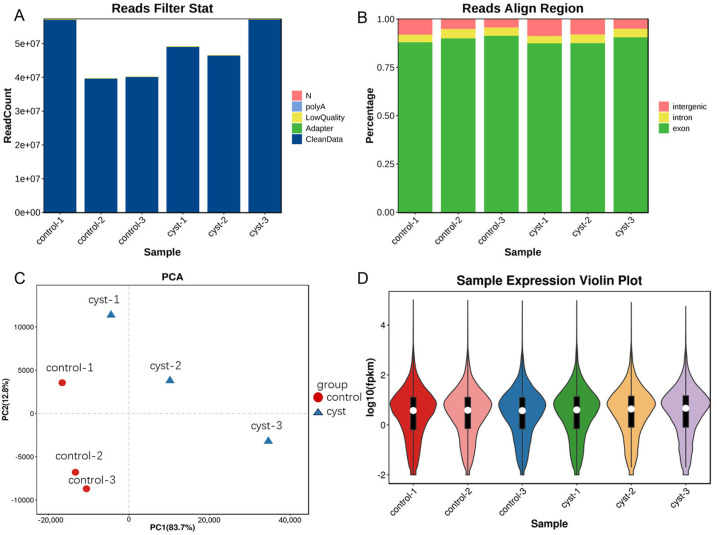
Characteristics of the sequencing data, quality control, and gene expression analysis. (**A**) Preprocessing of raw reads. (**B**) Regions of the reference genome to which the clean reads mapped, pink is intergenic, yellow is intron, green is exon. (**C**) Representative principal component analysis (PCA). (**D**) Distribution of gene expression in the samples, red is control-1, pink is control−2, blue is control−3, green is cyst−1, yellow is cyst−2, purple is cyst−3.

Transcriptome analyses were performed on normal and cystic luteal granulosa cells to identify the genes related to the development of luteal cysts. We obtained 57,229,092, 39,761,374, and 40,244,178 raw reads from the corpus luteum samples, and 49,227,446, 46,621,552, and 57,313,272 raw reads from the corpus luteum cysts (Figure 2A). The raw data were quality tested using FastQC [15]. For all of the samples, the Q20 values were >95%, indicating that the sequencing data were of reliable quality with a low error rate. After obtaining high-quality clean reads (Supplementary Table S2), HISAT2 was used to map the sequencing data to the reference genome [16]. The comparison rate of all the processed data was >95% (Supplementary Table S3), indicating that the sequence quality was sufficient for subsequent gene expression analysis (Figure 2B). More than 85% of the sequencing data mapped to the exon regions of the genes and other reads mapped to the intron or intergenic regions, further indicating the high quality of the sequencing data. The principal component analysis (PCA) results of the normal and cystic luteal cells are shown in Figure 2C; PC1 was 83.7% and PC2 was 12.8%. The degree of separation between PC1 and PC2 in the scatter plot indicated significant differences between the samples. The distribution trend of the gene expression was consistent among the cystic and normal corpus luteum samples, and the log10 of the Fragments Per Kilobase of the transcript per Million mapped reads (FPKM) values were between −2 and 2 (Figure 2D). All of the raw sequence data have been deposited in the China National Gene Bank (CNGB) Nucleotide Sequence Archive (CNSA) (https://db.cngb.org/cnsa/, accessed on 3 April 2022) under accession number CNP0004552.

### 3.2. Identification and Functional Annotation of Differentially Expressed Genes

**Figure 3:**
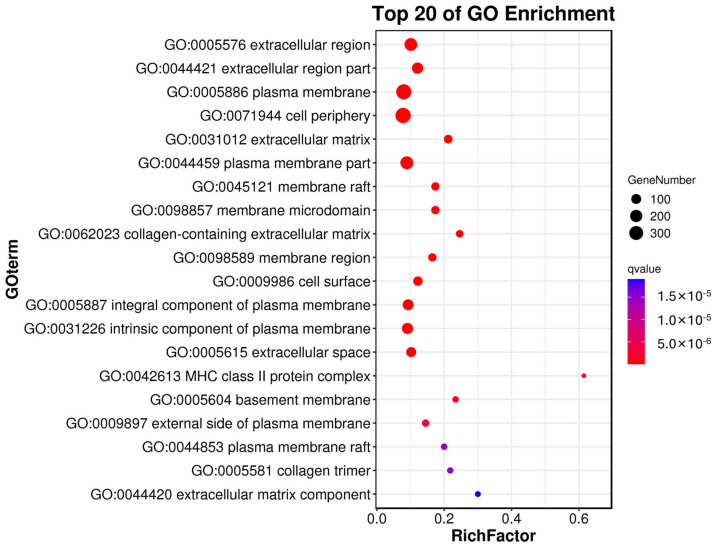
The top 20 most significantly enriched Gene Ontology (GO) terms. GO functional annotation of the differentially expressed genes. Distribution of the significantly enriched GO terms in the cellular component and molecular function categories.

We identified 1048 DEGs between the normal corpus luteum and cystic corpus luteum granulosa cells; 892 genes were significantly up−regulated, and 156 genes were significantly down-regulated. To predict the functions of the DEGs, we used GO and KEGG enrichment. The GO analysis showed that 558 terms were significantly enriched (*p* < 10^−5^); 527, 23, and 8 terms were under the biological process, cellular component, and molecular function categories, respectively. The top 20 most significantly enriched GO terms are shown in Figure 3. Many of these terms are associated with immune processes, including the immune system process, regulation of immune system process, immune response, immune effector process, regulation of immune system process, leukocyte activation, innate immune response, positive regulation of immune system process, and lymphocyte activation.

**Figure 4:**
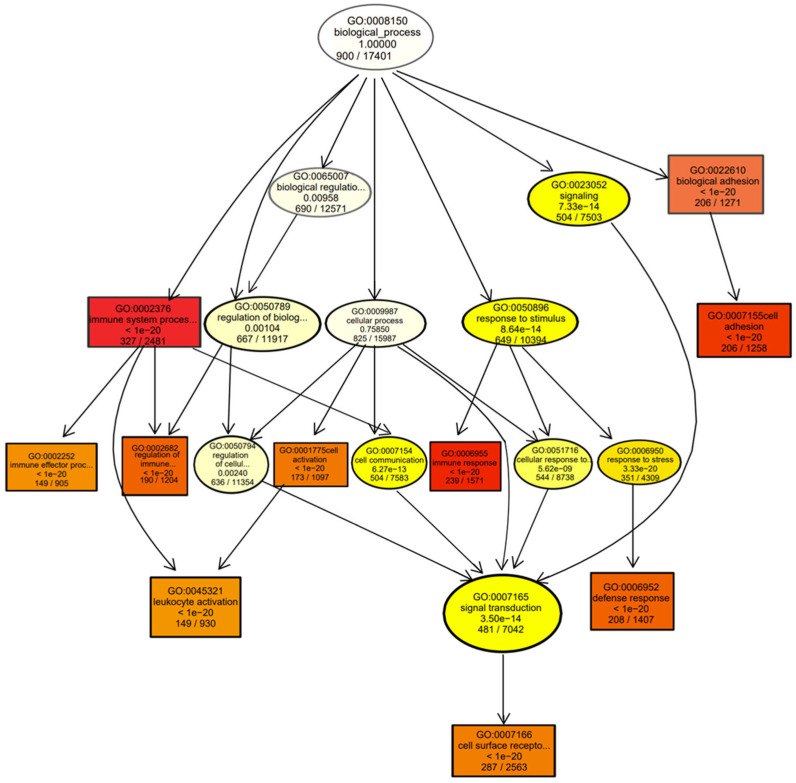
Directed acyclic graph of highly enriched Gene Ontology (GO) terms showing the hierarchical relationship of each node.

The top 10 most significantly enriched GO terms were selected as the main nodes of a directed acyclic graph (Figure 4), and the hierarchical relationship of each node was analyzed to determine the overlap of genes in different GO categories. Analysis of the directed acyclic graph showed that the DEGs affected the occurrence of corpus luteum cysts mainly by activating the immune system or by regulating the biological adhesion process. The activation of the immune system further promoted the leukocyte activation process and activated the immune response. These two biological processes were regulated through cell surface receptor signaling.

**Figure 5:**
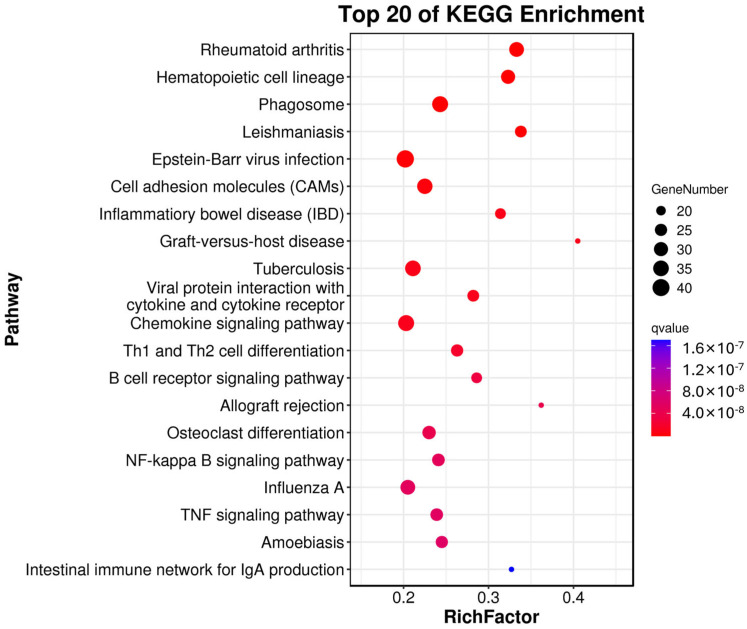
Kyoto Encyclopedia of Genes and Genomes (KEGG) pathway enrichment analysis of the differentially expressed genes.

The KEGG analysis showed that 79 pathways were significantly enriched. The top 20 significantly enriched pathways were classified into five main categories: cellular processes, metabolism, environmental information processing, human diseases, and organismal systems (Figure 5). In total, 10 of the top 20 pathways were related to diseases that mostly involve tumor formation, including cell adhesion molecules, Th1 and Th2 cell differentiation, the chemokine signaling pathway, the B cell receptor signaling pathway, the tumor necrosis factor signaling pathway, the NF−kappa B signaling pathway, and the retinol signaling pathway.

### 3.3. Genes Related to Luteal Cysts Identified by Transcriptome Data Analysis

Fifty of the significantly enriched KEGG pathways were selected for further analysis, and the genes involved in these pathways were sorted according to their fold change (Supplementary Table S4). The selected genes were mainly involved in pathways in cancer, cell adhesion molecules, cytokine-cytokine receptor interaction, transcriptional mis-regulation in cancers, the MAPK signaling pathway, the PI3K-Akt signaling pathway, the cAMP signaling pathway, and neuroactive ligand-receptor interaction. Among them, cell adhesion molecules are closely related to the GO terms that relate to the biological adhesion process. Further analysis of the DEGs (fold change > 2) related to cell adhesion molecules identified *ICOS*, *CD8B*, *PDCD1*, *CD2*, *SIGLEC1*, *CD22*, and *CD80*. These genes play important roles in stimulating the immune response processes and are candidate marker genes for the early detection of luteal cysts.

### 3.4. Metabolome Analysis and Principal Component Analysis

Metabolites in the cystic and normal corpus luteum were detected through gas chromatography-mass spectrometry in the positive and negative ion modes to ensure that the metabolite coverage rate was high and the detection was effective. We identified 22,622 original data peaks. After bias filtering, missing value recoding, and data standardization, 22,622 peaks were retained (Table 1), and 1106 metabolites were annotated.

**Figure 6:**
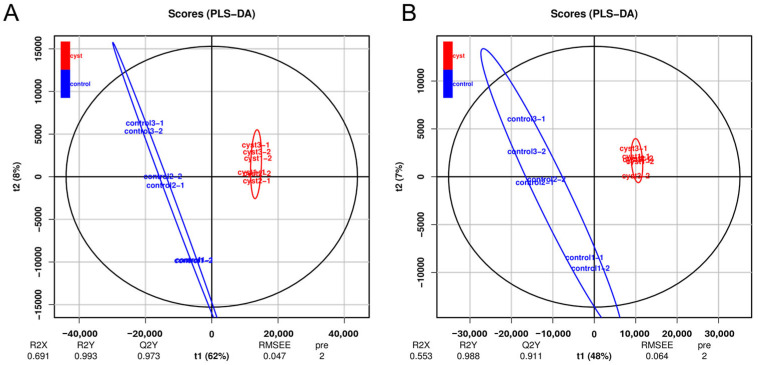
Principal component analyses of metabolic profiles were obtained using the positive ion mode, POS (**A**) and negative ion mode, NEG (**B**) models.

The PCA of the metabolic profiles of the differentially treated samples (Figure 6) showed obvious quadrantal differences in the metabolic patterns on the PC1 axis. After 200 permutations of the cystic and normal corpus luteum, different random R2 and Q2 values were obtained. R2Y and Q2Y were used to evaluate the validity of the model, and orthogonal projections to latent structures discriminant analysis (OPLS−DA) of PC1 and PC2 were completed. The scoring charts showed that each stage was clearly divided (Figure 6), indicating that cystic and normal corpus luteum have different metabolic patterns, and may therefore be physiologically different. 

### 3.5. KEGG Annotation of Differentially Abundant Metabolites in the Metabolome

**Figure 7:**
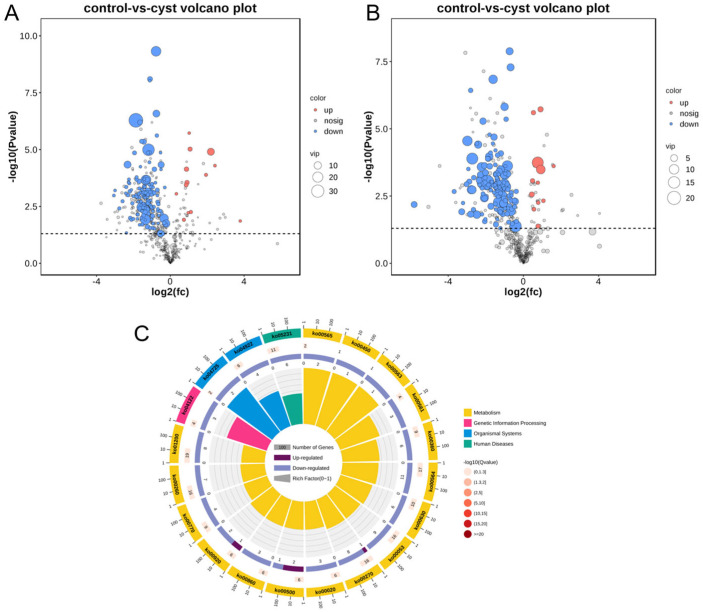
Volcano plots of differentially abundant metabolites between cyst and normal corpus luteum in positive ion mode, POS (**A**) and in negative ion mode, NEG (**B**) modes. Circular plot of KEGG orthology enrichment of differentially abundant metabolites (**C**).

The analysis of the differential metabolism showed that the number of down-regulated metabolites was significantly higher than the number of up−regulated metabolites in the cystic corpus luteum compared with the normal corpus luteum (Figure 7A,B). The genes in the pathways that were mapped and enriched by the differentially abundant metabolites were annotated with KEGG pathways (Figure 7C). The top 20 enriched pathways were mainly related to metabolic and genetic information processing diseases. Among them, seven significantly enriched pathways were identified, namely glycerophospholipid metabolism, cysteine and methionine metabolism, ascorbate and aldarate metabolism, glyoxylate and dicarboxylate metabolism, phenylalanine metabolism, glucagon signaling pathway, and choline metabolism in cancer.

### 3.6. Genes Related to Luteal Cyst Occurrence Based on the Metabolome

We focused on choline metabolism in the cancer pathway based on the location of the differentially abundant metabolites in the metabolic pathways. The cancer pathway is associated with the PI3K-Akt and MAPK signaling pathways, which is consistent with the results from the transcriptome analysis. These findings indicate that choline metabolism in the luteal cyst tissues is related to regulation of the MAPK and PI3K−Akt signaling pathways. *CSF3*, *COL1A1*, *PIK3R5*, *COL9A2*, and *COL1A2* had high differential expression levels in the PI3K-Akt signaling pathway, whereas, in the MAPK signaling pathway, the expression of *MAP4K1* and *FLNC* were significantly increased (by at least four times).

### 3.7. Combined Metabolomic and Transcriptomic Analysis

To better understand the interaction between granulosa cell metabolites and genes in the formation of luteal cysts, we conducted KEGG common pathway enrichment analysis for the differentially abundant metabolites and DEGs. A total of 26 pathways with significant differences were enriched in the KEGG pathway map, such as pathways in cancer, metabolic pathways, ovarian steroidogenesis, sphingolipid metabolism, fatty acid metabolism, and cAMP signaling. Pathways in cancer are at the top of the list, and we speculate that this pathway plays a key role in the formation of luteal cysts.

### 3.8. Verification of the Expression of Selected Genes

**Figure 8:**
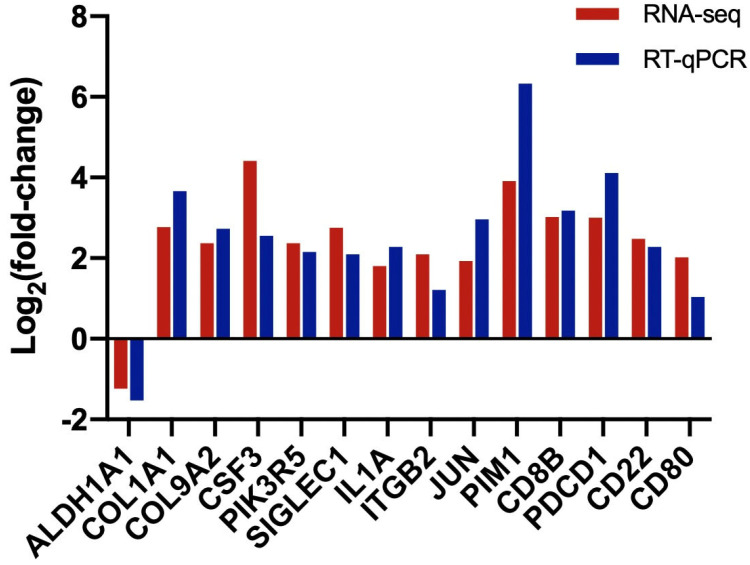
Verification of differentially expressed genes (DEG) expression by RT−qPCR. Aldehyde dehydrogenases 1 family member A1(ALDH1A1) gene; Collagen type I alpha 1 (COL1A1); Colony Stimulating Factor 3 (CSF3); Sialic Acid Binding Ig Like Lectin 1 (SIGLEC1); Interleukin 1 Alpha (IL1A); Integrin Subunit Beta 2(ITGB2); (Jun Proto−Oncogene) JUN; Pim1 Proto−Oncogene, Se −ine/Threonine Kinase (PIM1); CD8 Subunit Beta (CD8B); Programmed Cell Death 1 (PDCD1); CD22 Molecule (CD22); CD80 Molecule (CD80).

We selected the DEGs related to luteal cyst occurrence and verified their expression through RT−qPCR. The expression levels of the DEGs with specific functions were consistent with the RNA sequencing data (Figure 8).

### 3.9. SNPs in DEGs and Their Effects on Gene Expression

We detected 433,755 SNPs, 15,206 deletions, and 31,041 insertions in the DEGs (Supplementary File S1: snp. annot). These variants were located in 38 non-synonymous sites in the DEG sequences (Supplementary File S2: non-synonymous.snp.txt), including in five adhesion−related genes (*CD22*, *CD8B*, *CD80*, *PDCD1*, and *SIGLEC1*), two PI3K−Akt pathway genes (*PIK3R5*, *COL9A2*), and *FLNC* in the MAPK pathway. Combining these findings with the SNP variant results (Table 2), we identified these eight genes to be key in luteal cysts occurrence. We consider these genes to be candidate markers for the detection of luteal cysts because the pathways that these genes are involved in were identified as key pathways for the formation of luteal cysts.

## 4. Discussion

Most of the sequencing reads had Q20s > 95%, and the qPCR verification showed that the sequencing results were reliable. The DEGs were annotated, with functionally enriched GO terms and KEGG pathways, and a large number of pathways and GO terms related to luteal cysts were identified. The predicted biological processes and pathways will help to further the understanding of the regulatory mechanisms underlying luteal cysts.

Luteal cysts can affect normal estrus in sows [17]. In this study, many of the top 20 functionally enriched GO terms assigned to the DEGs in luteal cysts were genes involved in immune-related biological processes, including *SIGLEC1*, *PDCD1*, *ICOS*, and *MHCII*, and to significantly up-regulated genes enriched in cell adhesion molecule signaling pathways. The increased expression of *SIGLEC1* is closely related to tumor formation and metastasis [18,19,20], and the increased expression of *ICOS* leads to poor prognosis and significantly increased *PDCD1* expression in ovarian tumors [21,22,23]. The increased expression of these immune-related genes may promote the proliferation and differentiation of granulosa cells and the formation of cysts. The increased expression of *MHCII* enhances the recognition of tumors by the immune system and enhances anti-tumor immunity [24,25], thereby promoting a good prognosis [26,27]. These results indicate that substantial inflammatory and anti-inflammatory reactions occur in the luteal cells of cysts.

The KEGG enrichment showed that the DEGs of luteal granulosa cells are related to tumor occurrence involving the PI3K/Akt, JAK/STAT, and MAPK signaling pathways. PIK3C promotes the transmission of PI3K signaling and stimulates the formation of tumors [28,29,30], while *PIK3R5* is highly expressed in tumors [31,32]. *FGF* and *FGFR* are up-regulated in various tumor tissues [33,34], and FGFR4 activates a downstream oncogenic signaling pathway to promote cancer development [35,36,37]. Interleukins play important roles in the immune response [38,39]. *IL6* expression is significantly increased in tumor cells and *IL6* is used as a marker of advanced colorectal cancer [40,41,42,43]. The expression of *IL4R* is also up-regulated in various tumors [44,45,46]. *CSF1* expression is significantly increased in hepatocellular carcinoma and is positively correlated with tumor severity [47,48,49,50]. The expression of CSF1R in colorectal cancer tissues is significantly higher than that in paracancerous tissues [51]. These enriched tumor-related signaling pathways may play a key role in the regulation of luteal cyst development. Because immunity can both inhibit and promote the occurrence of tumors [52], it has been speculated that cysts may belong to a special state of tumor pathology. These findings indicate that exploring changes in the microenvironment of cystic luteal cells from the perspective of immunology and tumor occurrence can guide the analysis of luteal cyst occurrence and prognosis.

Aldehyde dehydrogenase (ALDH) oxidizes retinol, the main form of vitamin A, to retinal, and then irreversibly oxidizes retinal to retinoic acid. This metabolic process avoids the cytotoxicity of retinal [53]. Moreover, retinoic acid and cellular retinoic acid-binding protein 1 (CRABP1) are involved in the regulation of gene expression, which can inhibit tumor formation [54,55]. Significantly reduced ALDH expression decreases retinoic acid synthesis and impairs the retinoic acid signaling pathway, leading to glioblastoma. Moreover, in *Helicobacter pylori*-induced gastric cancer, the expression of ALDH1 and RALDH2 in the retinol metabolic pathway are decreased [56], and the intestinal retinoic acid level in a colorectal cancer mouse model was significantly decreased [57]. Granulosa cells are the main cells responsible for retinol uptake and metabolism to retinoic acid in the ovary [58,59]. In this study, the retinol metabolism pathway was one of the most significantly enriched pathways in granulosa cells of luteum cysts, and the expression of genes that encode key enzymes in this pathway, such as retinol dehydrogenase and ALDH1A, was significantly reduced. Therefore, we hypothesized that retinoic acid production was blocked in the granulosa cells of the corpus luteum, which disabled the retinol metabolism pathway, resulting in the abnormal degeneration of the corpus luteum and the formation of corpus luteum cysts.

Abnormal lipid uptake, synthesis, and anabolism are closely related to the malignant transformation of tumor cells [60,61]. Glycerophospholipids, the basic skeleton of biofilm systems, participate in transcription, energy metabolism, and signal transduction [62]. The follicular fluid of polycystic follicle syndrome has a decreased abundance of glycerol phospholipids [63], which affects follicular development and reduces the rate of insemination [64,65]. Phosphatidylcholine metabolism is also abnormal in polycystic follicle syndrome, which affects cell proliferation and differentiation [66,67]. These lipids are closely related to follicle development and the ability of the ovaries to respond to gonadotropins [68]. In this study, the metabolomics profiling of the luteal granulosa cells showed that the levels of glycerophospholipids and phosphocholine were significantly reduced, which may inhibit the response of luteal granulosa cells to gonadotropin signals [69]. We speculate that changes in the microenvironment that affect lipid metabolism may lead to the occurrence of luteum cysts.

## 5. Conclusions

The formation of luteal cysts is closely related to cancer- and immune-related signaling pathways. Cell adhesion molecules such as CD22, CD8B, CD80, PDCD1, SIGLEC1, PIK3R5, COL9A2, and FLNC can be used as markers for the initial identification of luteal cysts. Abnormal lipid metabolism is also involved in the formation of luteal cysts. Our results provide a new entry point for exploring the pathogenesis of porcine corpus luteum cysts.

## Figures and Tables

**Table 1:** Metabolite detection in luteum tissues by gas chromatography-mass spectrometry.

Type	All	Keep	Known	Unknown
POS ^1^	10,079	10,079	626	9453
NEG ^2^	12,543	12,543	480	12,063
Total	22,622	22,622	1106	21,516

^1^ POS means positive ion mode. ^2^ NEG means negative ion mode.

**Table 2:** Number of non-synonymous variants in eight candidate marker genes.

Gene	Number
*FLNC*	4
*PDCD1*	4
*COL9A2*	3
*CD22*	4
*CD8B*	3
*PIK3R5*	4
*CD80*	1
*SIGLEC1*	15

## Data Availability

Not applicable.

## Supplementary Materials

The following supporting information can be downloaded at: https://www.mdpi.com/article/10.3390/genes14101848/s1, Table S1: The names and sequences of 15 genes; Table S2: Data filtering statistics table; Table S3: Comparison of numbers with reference statistics; Table S4: control-vs-Cyst Pathway Enrichment; Supplementary File S1: snp. annot; Supplementary File S2: non-synonymous.snp.txt.

## References

[1] Tummaruk P., Kesdangsakonwut S., Kunavongkrit A. (2009). Relationships among specific reasons for culling, reproductive data, and gross morphology of the genital tracts in gilts culled due to reproductive failure in Thailand. Theriogenology.

[2] Szulańczyk-Mencel K., Rzasa A., Bielas W. (2010). Relationships between ovarian cysts and morphological and hormonal state of ovarian cortex in sows. Anim. Reprod. Sci..

[3] Yücel-Tenekeci G., Sepici-Dinçel A., Özkul İ.A. (2022). Pathomorphological Lesions in the Ovaries of Water Buffaloes. Acta Sci. Vet..

[4] Tummaruk P., Kesdangsakonwut S. (2012). Factors affecting the incidence of cystic ovaries in replacement gilts. Comp. Clin. Pathol..

[5] Amweg A.N., Salvetti N.R., Stangaferro M.L., Paredes A.H., Lara H.H., Rodríguez F.M., Ortega H.H. (2013). Ovarian localization of 11β-hydroxysteroid dehydrogenase (11βHSD): Effects of ACTH stimulation and its relationship with bovine cystic ovarian disease. Domest. Anim. Endocrinol..

[6] Vanholder T., Opsomer G., de Kruif A. (2006). Aetiology and pathogenesis of cystic ovarian follicles in dairy cattle: A review. Reprod. Nutr. Dev..

[7] Marelli B.E., Diaz P.U., Salvetti N.R., Rey F., Ortega H.H. (2014). mRNA expression pattern of gonadotropin receptors in bovine follicular cysts. Reprod. Biol..

[8] Salvetti N.R., Alfaro N.S., Velázquez M.M., Amweg A.N., Matiller V., Díaz P.U., Ortega H.H. (2012). Alteration in localization of steroid hormone receptors and coregulatory proteins in follicles from cows with induced ovarian follicular cysts. Reproduction.

[9] Zulu V.C., Sawamukai Y., Nakada K., Moriyoshi M. (2002). Relationship among insulin-like growth factor-I, blood metabolites and postpartum ovarian function in dairy cows. J. Vet. Med. Sci..

[10] Fortin C.S., Leader A., Mahutte N., Hamilton S., Léveillé M.C., Villeneuve M., Sirard M.A. (2019). Gene expression analysis of follicular cells revealed inflammation as a potential IVF failure cause. J. Assist. Reprod. Gen..

[11] Yao Z., You F.M., N’Diaye A., Knox R.E., McCartney C., Hiebert C.W., Pozniak C., Xu W. (2020). Evaluation of variant calling tools for large plant genome re-sequencing. BMC Bioinform..

[12] Hu G., Yue X., Song J., Xing G., Chen J., Wang H., Su N., Cui J. (2021). Calcium Positively Mediates Blue Light-Induced Anthocyanin Accumulation in Hypocotyl of Soybean Sprouts. Front. Plant Sci..

[13] Want E.J., Masson P., Michopoulos F., Wilson I.D., Theodoridis G., Plumb R.S., Shockcor J., Loftus N., Holmes E., Nicholson J. (2013). Global metabolic profiling of animal and human tissues via UPLC-MS. Nat. Protoc..

[14] Fu Y.P., Li C.Y., Peng X., Wangensteen H., Inngjerdingen K.T., Zou Y.F. (2022). Pectic polysaccharides from Aconitum carmichaelii leaves protects against DSS-induced ulcerative colitis in mice through modulations of metabolism and microbiota composition. Biomed. Pharmacother.

[15] Agarwal D., Kuhns R., Dimitriou C.N., Barlow E., Wahlin K.J., Enke R.A. (2022). Bulk RNA sequencing analysis of developing human induced pluripotent cell-derived retinal organoids. Sci. Data.

[16] Ding N., Yuan Z., Zhang X., Chen J., Zhou S., Deng Y. (2020). Programmable cross-ribosome-binding sites to fine-tune the dynamic range of transcription factor-based biosensor. Nucleic Acids Res..

[17] Toriumi H., Tsumagari S., Kuwahara Y., Ichikawa Y., Takeishi M., Sakai T. (2003). Development of a method of diagnosing ovarian disorders in sows and gilts using uterine ultrasonography. J. Vet. Med. Sci..

[18] Briem O., Källberg E., Kimbung S., Veerla S., Stenström J., Hatschek T., Hagerling C., Hedenfalk I., Leandersson K. (2023). CD169^+^ Macrophages in Primary Breast Tumors Associate with Tertiary Lymphoid Structures, T_regs_ and a Worse Prognosis for Patients with Advanced Breast Cancer. Cancers.

[19] Cassetta L., Fragkogianni S., Sims A.H., Swierczak A., Forrester L.M., Zhang H., Soong D.Y.H., Cotechini T., Anur P., Lin E.Y. (2019). Human tumor-associated macrophage and monocyte transcriptional landscapes reveal cancer-specific reprogramming, biomarkers, and therapeutic targets. Cancer Cell.

[20] Yu Y., Peng W. (2023). Recent progress in targeting the sialylated glycan-SIGLEC axis in cancer immunotherapy. Cancer Biol. Med..

[21] Faget J., Sisirak V., Blay J.Y., Caux C., Bendriss-Vermare N., Ménétrier-Caux C. (2013). ICOS is associated with poor prognosis in breast cancer as it promotes the amplification of immunosuppressive CD4^+^ T cells by plasmacytoid dendritic cells. Onocimmunocogy.

[22] Amatore F., Gorvel L., Olive D. (2020). Role of Inducible Co-Stimulator (ICOS) in cancer immunotherapy. Expert. Opin. Biol. Ther..

[23] Zhao X., Wang Y., Jiang X., Mo B., Wang C., Tang M., Rong Y., Zhang G., Hu M., Cai H. (2023). Comprehensive analysis of the role of ICOS (CD278) in pan-cancer prognosis and immunotherapy. BMC Cancer.

[24] Accolla R.S., Ramia E., Tedeschi A., Forlani G. (2019). CIITA-driven MHC class II expressing tumor cells as antigen presenting cell performers: Toward the construction of an optimal anti-tumor vaccine. Front. Immunol..

[25] Chatterjee F., Spranger S. (2023). MHC-dressing on dendritic cells: Boosting anti-tumor immunity via unconventional tumor antigen presentation. Seminars in Immunology.

[26] Thomas P., Srivastava S. (2022). MHC-II molecules present RhoC-derived peptides on the surface of tumour cells. bioRxiv.

[27] Jiang X., Ying Q., Xia W., Li J., Shi N., Feng Q., Tang A., Yi X. (2022). An immune-lncRNA risk model to predict prognosis for patients with head and neck squamous cell carcinoma. bioRxiv.

[28] Samuels Y., Diaz L.A., Schmidt-Kittler O., Cummins J.M., Delong L., Cheong I., Rago C., Huso D.L., Lengauer C., Kinzler K.W. (2005). Mutant PIK3CA promotes cell growth and invasion of human cancer cells. Cancer Cell.

[29] Hoxhaj G., Manning B.D. (2020). The PI3K–AKT network at the interface of oncogenic signalling and cancer metabolism. Nat. Rev. Cancer.

[30] Ren A.A., Snellings D.A., Su Y.S., Hong C.C., Castro M., Tang A.T., Detter M.R., Hobson N., Girard R., Romanos S. (2021). PIK3CA and CCM mutations fuel cavernomas through a cancer-like mechanism. Nature.

[31] Paula L.M., Moraes L.H.F., Canto A.L., Dos Santos L., Martin A.A., Rogatto S.R., De Azevedo Canevari R. (2017). Analysis of molecular markers as predictive factors of lymph node involvement in breast carcinoma. Oncol. Lett..

[32] Li L.Y., Kim H.J., Park S.A., Lee S.H., Kim L.K., Lee J.Y., Kim S., Kim Y.T., Kim S.W., Nam E.J. (2019). Genetic Profiles Associated with Chemoresistance in Patient-Derived Xenograft Models of Ovarian Cancer. Cancer Res. Treat..

[33] Shin W.S., Lee H.W., Lee S.T. (2019). Catalytically inactive receptor tyrosine kinase PTK7 activates FGFR1 independent of FGF. Faseb J..

[34] Gammelgaard K.R., Vad-Nielsen J., Clement M.S., Weiss S., Daugaard T.F., Dagnæs-Hansen F., Meldgaard P., Sorensen B.S., Nielsen A.L. (2019). Up-regulated FGFR1 expression as a mediator of intrinsic TKI resistance in EGFR-mutated NSCLC. Transl. Oncol..

[35] Chen T., Liu H., Liu Z., Li K., Qin R., Wang Y., Liu J., Li Z., Gao Q., Pan C. (2021). FGF19 and FGFR4 promotes the progression of gallbladder carcinoma in an autocrine pathway dependent on GPBAR1-cAMP-EGR1 axis. Oncogene.

[36] Wang L., Ren Z., Yu B., Tang J. (2021). Development of nomogram based on immune-related gene FGFR4 for advanced non-small cell lung cancer patients with sensitivity to immune checkpoint inhibitors. J. Transl. Med..

[37] Shiu B., Hsieh M.H., Ting W., Chou M., Chang L., Huang C., Su S., Yang S. (2021). Impact of FGFR4 gene polymorphism on the progression of colorectal cancer. Diagnostics.

[38] Briukhovetska D., Dörr J., Endres S., Libby P., Dinarello C.A., Kobold S. (2021). Interleukins in cancer: From biology to therapy. Nta Rev. Cancer.

[39] She Y.X., Yu Q.Y., Tang X.X. (2021). Role of interleukins in the pathogenesis of pulmonary fibrosis. Cell Death Discov..

[40] Tawara K., Scott H., Emathinger J.M., Wolf C.L., La Joie D., Hedeen D.S., Bond L., Montgomery P.G., Jorcyk C.L. (2019). HIGH expression of OSM and IL-6 are associated with decreased breast cancer survival: Synergistic induction of IL-6 secretion by OSM and IL-1β. Oncotarget.

[41] Wu J., Gao F., Wang C., Qin M., Han F., Xu T., Hu Z., Long Y., He X., Deng X. (2019). IL-6 and IL-8 secreted by tumour cells impair the function of NK cells via the STAT3 pathway in oesophageal squamous cell carcinoma. JECCR.

[42] Chonov D.C., Ignatova M.M.K., Ananiev J.R., Gulubova M.V. (2019). IL-6 activities in the tumour microenvironment. Part 1. Open Access Maced. J. Med. Sci..

[43] Goulet C.R., Champagne A., Bernard G., Vandal D., Chabaud S., Pouliot F., Bolduc S. (2019). Cancer-associated fibroblasts induce epithelial–mesenchymal transition of bladder cancer cells through paracrine IL-6 signalling. BMC Cancer.

[44] Gunassekaran G.R., Hong C.M., Vadevoo S.M., Chi L., Guruprasath P., Ahn B.C., Kim H., Kang T.H., Lee B. (2018). Non-genetic engineering of cytotoxic T cells to target IL-4 receptor enhances tumor homing and therapeutic efficacy against melanoma. Biomaterials.

[45] Chi L., Na M.H., Jung H.K., Vadevoo S.M., Kim C., Padmanaban G., Park T.I., Park J., Hwang I., Park K.U. (2015). Enhanced delivery of liposomes to lung tumor through targeting interleukin-4 receptor on both tumor cells and tumor endothelial cells. J. Control Release.

[46] Murugan P.V.S., Rangaswamy G.G., Lee B. (2022). Interleukin-4 receptor-targeted Abraxane inhibits tumor growth by enhancing drug delivery and reprogramming of M2-type macrophages into M1 phenotype. Cancer Res..

[47] Wei C.Y., Zhu M.X., Zhang P.F., Huang X.Y., Wan J.K., Yao X.Z., Hu Z.T., Chai X.Q., Peng R., Yang X. (2022). PKCα/ZFP64/CSF1 axis resets the tumor microenvironment and fuels anti-PD1 resistance in hepatocellular carcinoma. J. Hepatol..

[48] Hu Z.Q., Zhou S.L., Li J., Zhou Z.J., Wang P.C., Xin H.Y., Mao L., Luo C.B., Yu S.Y., Huang X.W. (2020). Circular RNA sequencing identifies CircASAP1 as a key regulator in hepatocellular carcinoma metastasis. Hepatology.

[49] Guo X.Y., Zhang J.Y., Shi X.Z., Wang Q., Shen W., Zhu W.W., Liu L.K. (2020). Upregulation of CSF-1 is correlated with elevated TAM infiltration and poor prognosis in oral squamous cell carcinoma. Am. J. Transl. Res..

[50] Sebban S., Farago M., Rabinovich S., Lazer G., Idelchuck Y., Ilan L., Pikarsky E., Katzav S. (2014). Vav1 promotes lung cancer growth by instigating tumor-microenvironment cross-talk via growth factor secretion. Oncotarget.

[51] Li H., Tang S. (2021). Colony stimulating factor-1 and its receptor in gastrointestinal malignant tumors. J. Cancer.

[52] Peña-Romero A.C., Orenes-Piñero E. (2022). Dual effect of immune cells within tumour microenvironment: Pro-and anti-tumour effects and their triggers. Cancers.

[53] Kumar S., Sandell L.L., Trainor P.A., Koentgen F., Duester G. (2012). Alcohol and aldehyde dehydrogenases: Retinoid metabolic effects in mouse knockout models. Biochim. Biophys. Acta.

[54] Chen M.C., Hsu S.L., Lin H., Yang T. (2014). Retinoic acid and cancer treatment. Biomedicine.

[55] Petkovich M., Chambon P. (2022). Retinoic acid receptors at 35 years. J. Mol. Endocrinol..

[56] Epplein M., Signorello L.B., Zheng W., Cai Q., Hargreaves M.K., Michel A., Pawlita M., Fowke J.H., Correa P., Blot W.J. (2011). Helicobacter pylori prevalence and circulating micronutrient levels in a low-income United States population. Cancer Prev. Res..

[57] Bhattacharya N., Yuan R., Prestwood T.R., Penny H.L., Dimaio M.A., Reticker-Flynn N.E., Krois C.R., Kenkel J.A., Pham T.D., Carmi Y. (2016). Normalizing microbiota-induced retinoic acid deficiency stimulates protective CD8^+^ T cell-mediated immunity in colorectal cancer. Immunity.

[58] Cai S., Chen M., Xue B., Zhu Z., Wang X., Li J., Wang H., Zeng X., Qiao S., Zeng X. (2023). Retinoic acid enhances ovarian steroidogenesis by regulating granulosa cell proliferation and MESP2/STAR/CYP11A1 pathway. J. Adv. Res..

[59] Fonseca B.M., Cruz R., Pinto B., Costa L., Felgueira E., Oliveira P., Casal S., Rebelo I. (2022). Retinoic acid (*all-trans*) presents antioxidant properties within human ovary and reduces progesterone production by human granulosa cells. Syst. Biol. Reprod. Med..

[60] Cui M.Y., Yi X., Zhu D.X., Wu J. (2021). Aberrant lipid metabolism reprogramming and immune microenvironment for gastric cancer: A literature review. Transl. Cancer Res..

[61] Zhang J., Song Y., Shi Q., Fu L. (2021). Research progress on FASN and MGLL in the regulation of abnormal lipid metabolism and the relationship between tumor invasion and metastasis. Front. Med..

[62] Cheng H., Wang M., Su J., Li Y., Long J., Chu J., Wan X., Cao Y., Li Q. (2022). Lipid Metabolism and Cancer. Life.

[63] Guan S., Liu Y., Guo Y., Shen X.X., Liu Y., Jin H. (2022). Potential biomarkers for clinical outcomes of IVF cycles in women with/without PCOS: Searching with metabolomics. Front. Endocrinol..

[64] Bai Y., Zhang F., Zhang H., Xu C., Wu L., Xia C. (2020). Follicular Fluid Metabolite Changes in Dairy Cows with Inactive Ovary Identified Using Untargeted Metabolomics. Biomed. Res. Int..

[65] Xu C., Xia C., Sun Y., Xiao X., Wang G., Fan Z., Shu S., Zhang H., Xu C., Yang W. (2016). Metabolic profiles using 1H-nuclear magnetic resonance spectroscopy in postpartum dairy cows with ovarian inactivity. Theriogenology.

[66] Ridgway N.D. (2013). The role of phosphatidylcholine and choline metabolites to cell proliferation and survival. Crit. Rev. Biochem. Mol. Biol..

[67] Podo F., Paris L., Cecchetti S., Spadaro F., Abalsamo L., Ramoni C., Ricci A., Pisanu M.E., Sardanelli F., Canese R. (2016). Activation of phosphatidylcholine-specific phospholipase C in breast and ovarian cancer: Impact on MRS-detected choline metabolic profile and perspectives for targeted therapy. Front. Oncol..

[68] Santos P.H., Fontes P.K., Franchi F.F., Nogueira M.F., Belaz K.R., Tata A., Eberlin M., Sudano M.J., Barros C.M., Castilho A.C. (2017). Lipid profiles of follicular fluid from cows submitted to ovarian superstimulation. Theriogenology.

[69] Korbecki J., Bosiacki M., Gutowska I., Chlubek D., Baranowska-Bosiacka I. (2023). Biosynthesis and Significance of Fatty Acids, Glycerophospholipids, and Triacylglycerol in the Processes of Glioblastoma Tumorigenesis. Cancers.

